# Efficacy of armodafinil on reducing excessive sleepiness in patients with shift work disorder: A systematic review protocol

**DOI:** 10.1192/j.eurpsy.2021.2097

**Published:** 2021-08-13

**Authors:** J. Correia, P. Silva, F. Mendes, A.R. Ferreira, L. Fernandes

**Affiliations:** 1 Medical Student, Faculty of Medicine of Porto University, Porto, Portugal, Portugal; 2 Cintesis – Center For Health Technology And Services Research; Psychiatry Service Of Centro Hospitalar Universitário De São João; Department Of Clinical Neuroscience And Mental Health, Faculty of Medicine of Porto University, Porto, Portugal, Portugal

**Keywords:** Armodafinil, Shift Work Disorder, Sleepiness, Sistematic Review

## Abstract

**Introduction:**

Previous studies have demonstrated that night-shift work is associated with adverse effects impacting physical and psychological health, including the Shift Work Disorder (SWD). SWD is a circadian rhythm disorder characterized by sleepiness and insomnia, resulting from working a shift other than the traditional daytime-shift. Armodafinil, a modafinil longer-lasting R-isomer, is approved for SWD treatment. Due to its pharmacodynamic profile, it may result in more sustained wakefulness during night-shifts than Modafinil.

**Objectives:**

To conduct a systematic review and meta-analysis of randomized controlled trials (RCTs) comparing the efficacy of Armodafinil vs Modafinil and/or placebo on reducing SWD excessive sleepiness.

**Methods:**

Will follow PRISMA guidelines. A systematic search will be conducted on PubMed, Web of Science, Scopus and ClinicalTrials.gov databases. RCTs comparing Armodafinil with Modafinil and/or Placebo for SWD treatment will be included. No language nor date restrictions will be applied. Outcomes of interest are prespecified as follows: the primary endpoint will be objective sleepiness; secondary endpoints will include subjective sleepiness, adverse effects, awareness, reaction time, memory and cognition. Retrieved studies will be independently screened for eligibility by two reviewers. Disagreements will be solved by consensus or by a third reviewer. Primary studies methodological quality will be assessed and data extracted independently using a standardized extraction-form.

**Results:**

Data will be described and reported as narrative text and summary tables. Heterogeneity of the included studies will be assessed and, if possible, a meta-analysis will be conducted.

**Conclusions:**

It is expected that this systematic review and meta-analysis favours Armodafinil over Modafinil and placebo in the treatment of SWD.

**Disclosure:**

No significant relationships.

